# Supplementary material for: Shigella hijacks the exocyst to cluster macropinosomes for efficient vacuolar escape
Source: PLoS Pathog. 2020 Aug 31;16(8):e1008822. doi: 10.1371/journal.ppat.1008822 (PMC7485983; doi:10.1371/journal.ppat.1008822)
Supplement: S1 Table — (DOCX) [file ppat.1008822.s001.docx]

**S1 Table. Plasmids used in this study**

| Plasmid | Protein | Reference |
| --- | --- | --- |
| pOrange-Galectin3 | Galectin3-mOrange | (Ray et al., 2010) |
| pEGFP-Actin | Actin-eGFP | (Ehsani et al., 2012) |
| pEGFP-Sec3 | eGFP-Sec3 | Kind gift from Jacques Camonis |
| pEGFP-Sec5 | eGFP-Sec5 | Kind gift from Jacques Camonis |
| pEGFP-Sec8 | eGFP-Sec8 | Kind gift from Jacques Camonis |
| pEGFP-Sec15 | eGFP-Sec15 | Kind gift from Jacques Camonis |
| pEGFP-Exo70 | eGFP-Exo70 | Kind gift from Jacques Camonis |
| pmApple-Sec5 | mApple-Sec5 | In this study |
| pEGFP-Sec5-ΔCorEx | eGFP-Sec5-ΔCorEx | In this study |
| pEGFP-Exo70-ΔCorEx | eGFP-Exo70-ΔCorEx | In this study |
| mApple-Rab11A-7 | mApple-Rab11A | Kind gift from Michael Davidson (addgene #54942) |
| mApple-Rab11A-S25N | mApple-Rab11A-S25N | In this study |
| eGFP-Rab8A | eGFP-Rab8A | Kind gift from Arnaud Echard |
| eGFP-Rab8A-T22N | eGFP-Rab8A-T22N | Kind gift from Arnaud Echard |
| mApple-Rab8A | mApple-Rab8A | In this study |
| mApple-Rab8A-T22N | mApple-Rab8A-T22N | In this study |
