# Supplementary material for: Shigella hijacks the exocyst to cluster macropinosomes for efficient vacuolar escape
Source: PLoS Pathog. 2020 Aug 31;16(8):e1008822. doi: 10.1371/journal.ppat.1008822 (PMC7485983; doi:10.1371/journal.ppat.1008822)
Supplement: S2 Table — (DOCX) [file ppat.1008822.s002.docx]

**S2 Table. Primers for cloning**

| Name | Sequence |
| --- | --- |
| Exo70-dCorEx-For | AGTTATCCTGCCTGGACCATGTTATCA |
| Exo70-dCorEx-Rev | GCAGGATAACTTGTCCTCGATCTCCCGCC |
| Sec5-dCorEx-For | AAGGGTGGC CGGTTTAAATTTCTCTTCAACC |
| Sec5-dCorEx-Rev | GCAGGATAACTTGTCCTCGATCTCCCGCC |
| Sec5-EcoRI-For | GGGGGGAATTCATGTCTCGATCCCGGCAG |
| Sec5-NotI-Rev | GGGGGGCGGCCGCTTATCTAGATCCGGTGGATCC |
| mApple-Rab11-DN-For | TGTTGGAAAGA**A**TAATCTCCTG |
| mApple-Rab11-DN-Rev | TCCAACACCAGAATCTCCAATAAG |
