# Supplementary material for: Shigella hijacks the exocyst to cluster macropinosomes for efficient vacuolar escape
Source: PLoS Pathog. 2020 Aug 31;16(8):e1008822. doi: 10.1371/journal.ppat.1008822 (PMC7485983; doi:10.1371/journal.ppat.1008822)
Supplement: S3 Table — (DOCX) [file ppat.1008822.s003.docx]

**S3 Table. Primers for real-time quantitative PCR**

| Target | Species | Sequence |
| --- | --- | --- |
| Hprt (Control) | *Homo sapiens* | 5’-TTTGCTGACCTGCTGGATTAC-3’  5’-CAAGACATTCTTTCCAGTTAAAGTTG-3’ |
| Exo70 | *Homo sapiens* | 5’-AGCTTGACGAAGGCACTGAC-3’  5’-TTCCTCTGGGGTTCCCTACT-3’ |
| Sec5 | *Homo sapiens* | 5’-GAGATGCCGGTCACAAGG-3’  5’-AGTCTTCGGCGAGACCTACC-3’ |
