# Supplementary material for: Shigella hijacks the exocyst to cluster macropinosomes for efficient vacuolar escape
Source: PLoS Pathog. 2020 Aug 31;16(8):e1008822. doi: 10.1371/journal.ppat.1008822 (PMC7485983; doi:10.1371/journal.ppat.1008822)
Supplement: S4 Table — (DOCX) [file ppat.1008822.s004.docx]

**S4 Table. Some exocyst subunits and the regulatory GTPase are enriched at the IAMs (INF-M *vs* INF-NM).**

|  |  |  | **Label-free Quantification (LFQ) intensity** | | | | | | |  | |  | |
| --- | --- | --- | --- | --- | --- | --- | --- | --- | --- | --- | --- | --- | --- |
| **Protein** | **log_2_**  **Fold-change** | **Adjusted**  **p-value** | **INF-M-1** | **INF-M-2** | **INF-M-3** | **INF-NM-1** | **INF-NM-2** | **INF-NM-3** | **# Peptides** | | **Molecular Weight (kDa)** | |  |
| EXOC1 (Sec3) | NA | NA | 3.3E+07 | 3.4E+07 | 2.0E+07 | 0 | 0 | 0 | 7 | | 100.28 | |  |
| EXOC2 (Sec5) | 1.29 | 2.15E-04 | 3.8E+07 | 5.0E+07 | 0 | 1.6E+07 | 1.7E+07 | 0 | 10 | | 104.07 | |  |
| EXOC4 (Sec8) | 1.04 | 4.86E-04 | 3.3E+07 | 4.6E+07 | 2.2E+07 | 1.4E+07 | 2.3E+07 | 1.2E+07 | 15 | | 110.5 | |  |
| Rab8A | 1.11 | 4.91E-04 | 2.0E+08 | 9.9E+07 | 8.3E+07 | 7.2E+07 | 4.4E+07 | 5.1E+07 | 8 | | 23.668 | |  |

|  |  |  |  |  |  |
| --- | --- | --- | --- | --- | --- |
