# Supplementary material for: Shigella hijacks the exocyst to cluster macropinosomes for efficient vacuolar escape
Source: PLoS Pathog. 2020 Aug 31;16(8):e1008822. doi: 10.1371/journal.ppat.1008822 (PMC7485983; doi:10.1371/journal.ppat.1008822)
Supplement: S5 Table — (DOCX) [file ppat.1008822.s005.docx]

**S5 Table. The exocyst subunit and the regulatory GTPase are enriched at the IAMs (INF-M *vs* Ctrl-M).**

|  |  |  | **Label-free Quantification (LFQ) intensity** | | | | | | |  | |  | |
| --- | --- | --- | --- | --- | --- | --- | --- | --- | --- | --- | --- | --- | --- |
| **Protein** | **log_2_**  **Fold-change** | **Adjusted**  **p-value** | **INF-M-1** | **INF-M-2** | **INF-M-3** | **Ctrl-M-1** | **Ctrl-M-2** | **Ctrl-M-3** | **# Peptides** | | **Molecular Weight (kDa)** | |  |
| EXOC6 (Sec15) | NA | NA | 9.4E+06 | 9.7E+06 | 0 | 0 | 0 | 0 | 3 | | 81.851 | |  |
| RalA | NA | NA | 5.0E+07 | 5.7E+07 | 6.1E+07 | 0 | 0 | 0 | 6 | | 23.567 | |  |
