# Supplementary figures and images for: Shigella hijacks the exocyst to cluster macropinosomes for efficient vacuolar escape
Source: PLoS Pathog. 2020 Aug 31;16(8):e1008822. doi: 10.1371/journal.ppat.1008822 (PMC7485983; doi:10.1371/journal.ppat.1008822)

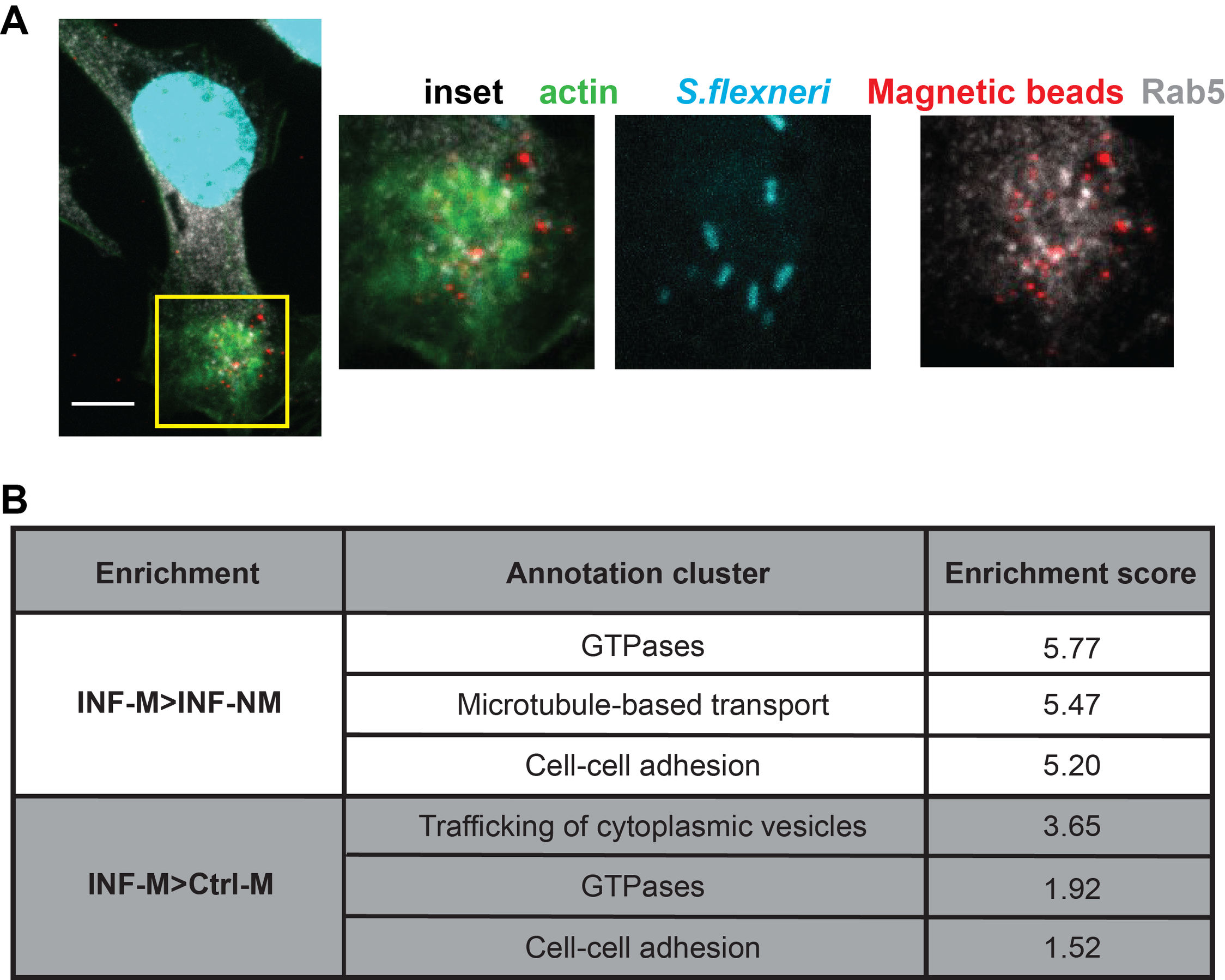

Supplement: S1 Fig — (A) Confocal image of z-projection of a representative infection focus of S. flexneri-infected HeLa in the presence of magnetic beads. Inset of the infection focus was highlighted in yellow and was shown, where S. flexneri was stained by DAPI (blue) and actin was stained by phalloidin (green). Immunofluorescence staining of endogenous Rab5A (in grey), a macropinosome marker, indicated that magnetic beads used in this study (in red) were present in Rab5A-positive IAMs. Scale bar is 10 μm. (B) Analysis of annotated clustering was performed by online software DAVID. The enriched clusters in the respective comparison were listed according to the descending order of the enrichment score. (TIF) [file ppat.1008822.s006.tif]

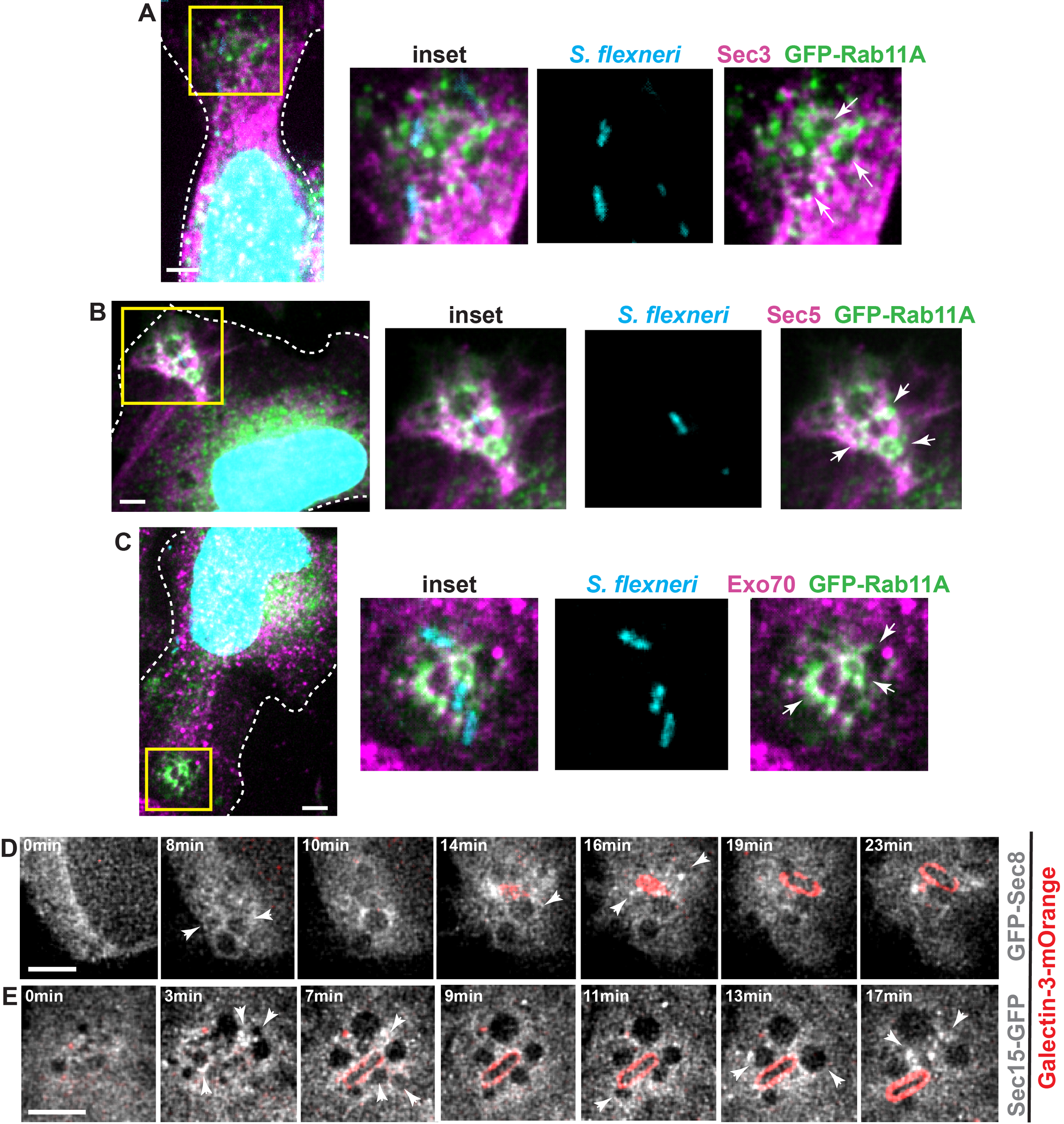

Supplement: S2 Fig — Confocal images of z-projection of representative infection foci of S. flexneri-infected HeLa cells. Insets of the infection foci were highlighted in yellow and were shown in A-C. Scale bars are 5 μm. Immunofluorescence staining with GFP-tagged Rab11A confirmed that endogenous (A) Sec3 (magenta), (B) Sec5 (magenta) and (C) Exo70 (magenta) were recruited to S. flexneri IAMs that are labelled by Rab11A (green) (as marked by the white arrows). Nuclei and S. flexneri were stained by DAPI (blue). Time-lapse microscopic analysis of the recruitment of GFP-tagged (D) Sec8 and (E) Sec15 (in grey) in the presence of Galectin-3-mOrange (red), a marker of vacuolar rupture. Images were recorded every minute and z-projections of representative entry sites are shown. The white arrowheads indicate the IAMs enriched with the respective exocyst subunits. Scale bars are 5 μm. (TIF) [file ppat.1008822.s007.tif]

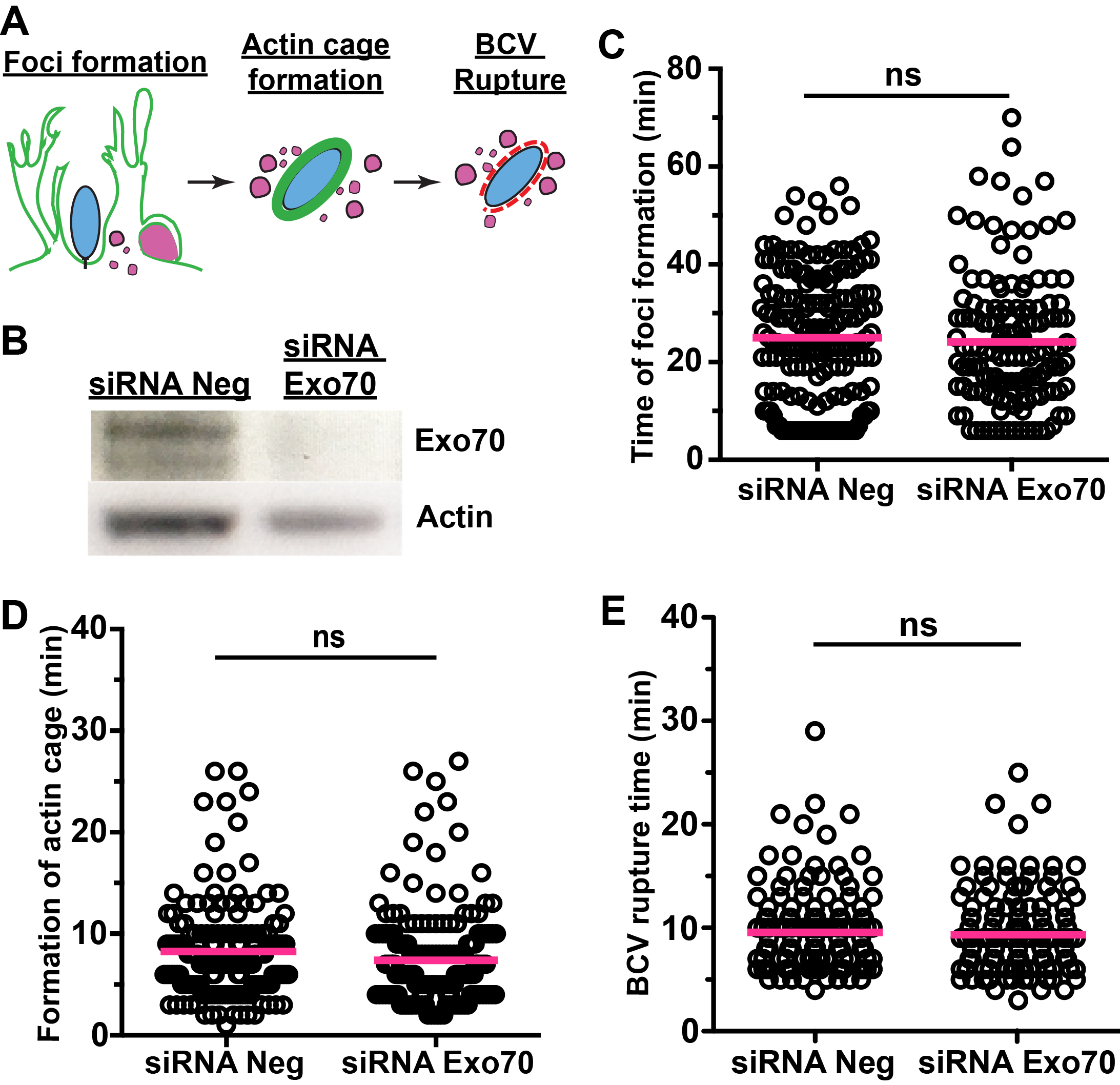

Supplement: S3 Fig — (A) Analysis of the timing of actin cage formation (green) and BCV rupture (dotted red ellipse) with reference to the onset of membrane ruffling during infection foci formation (black). (B) Knock-down efficiency of Exo70 by RNA interference (siRNA Exo70) was confirmed by Western blotting, whereas non-targeting RNA (siRNA Neg) was used as a control. Actin was used as the loading control of the Western blot. Time-lapse microscopic analyses of S. flexneri infection of control HeLa cells and Exo70-depleted HeLa cells were performed to examine the early entry steps of S. flexneri invasion as illustrated in (B), including (C) time of foci formation, (D) time of actin cage formation and (E) time of BCV rupture. At least a total of 135 infection foci (n > 135), 135 ruptured BCVs (n > 135) and 160 actin cages (n > 160) in triplicate experiments were analyzed in each condition. The bars (magenta) represent the mean and the unpaired t-tests were carried out (ns: non-significant). (TIF) [file ppat.1008822.s008.tif]

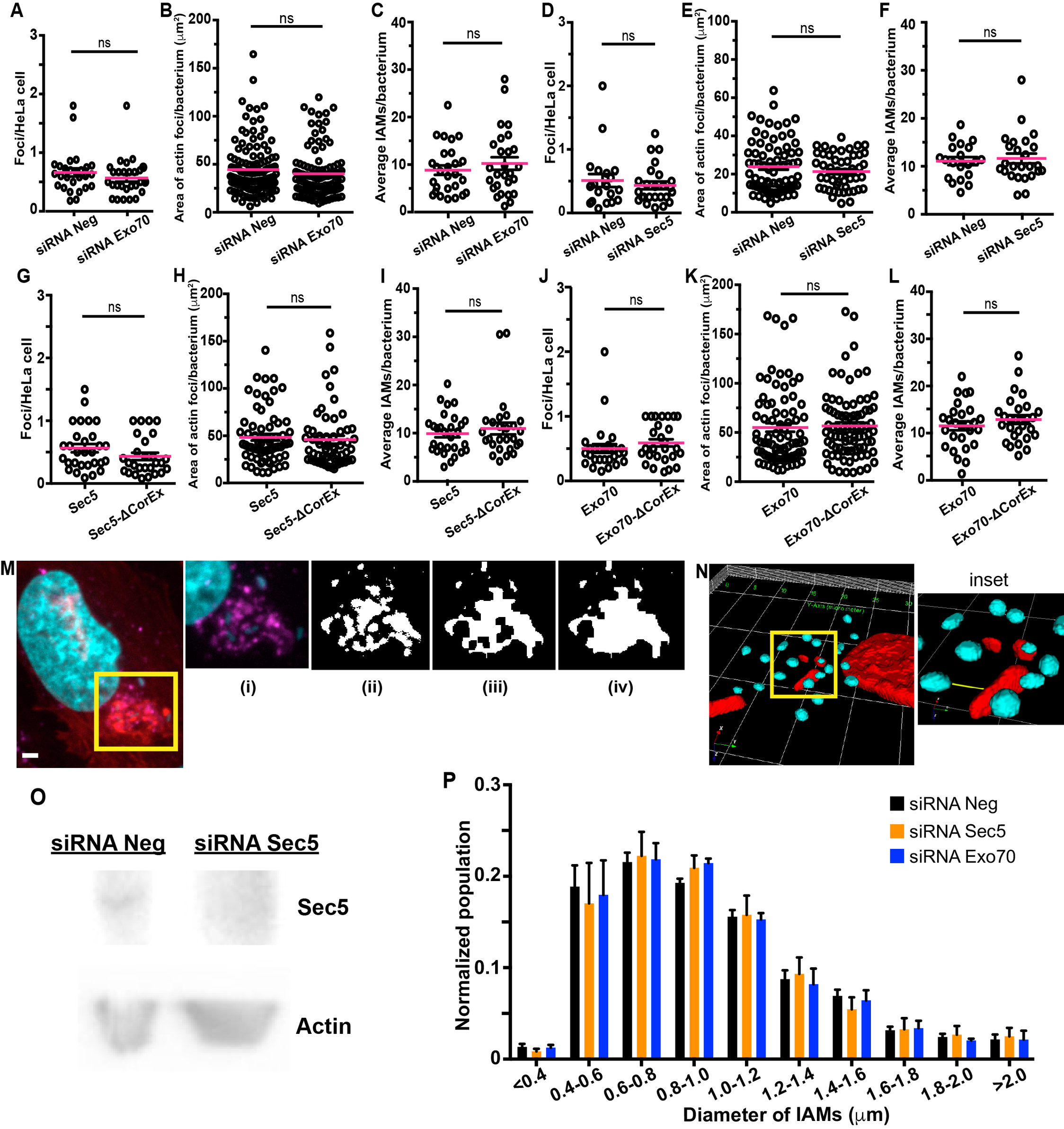

Supplement: S4 Fig — S. flexneri invasion of HeLa cells was performed in the presence of fluorescent dextran. The number of infection foci per HeLa cell, the area of the actin foci and the number of IAMs per S. flexneri were evaluated in different conditions including (A-C) RNA interference of non-targeting control (siRNA Neg) versus Exo70 depletion (siRNA Exo70), (D-F) RNA interference of non-targeting control (siRNA Neg) versus Sec5 depletion (siRNA Sec5), (G-I) expression of wild-type Sec5 or Sec5 lacking the core exocyst assembly motif (Sec5-ΔCorEx) and (J-L) expression of wild-type Exo70 or Exo70 lacking the core exocyst assembly motif (Exo70-ΔCorEx). At least 25 images consisting of more than 50 infection foci (n > 50) were analyzed in triplicates of each condition. The bars (magenta) represent the mean and unpaired t-tests were carried out (ns: non-significant). (M) Quantification of the areas occupied by IAMs per S. flexneri using software Image J. Confocal images of S. flexneri infection in the presence of fluorescent dextran (magenta) were analyzed. Nuclei and S. flexneri were stained by DAPI (blue) while the actin infection focus was marked by phalloidin (red). Scale bar is 5 μm. (i) Infection focus of S. flexneri was selected and the channel with IAM (marked by the fluorescent dextran) was segmented. (ii) Binary image of the dextran channel was obtained. (iii) Adjacent IAMs were connected to form a patch that outlined the clustered IAMs, while any empty spaces within the patch were filled. (iv) The area occupied by the IAMs at an individual infection focus was estimated by measuring the area bordering the patches of the clustered IAMs at the given focus using the built-in Analyze Particles function. (N) The spatial distribution of IAMs around S. flexneri at infection focus was examined in 3D using software Icy. Inset of the infection focus was highlighted in yellow. The distances between individual IAMs (in blue) and S. flexneri (in red) were estimated, which is marked by th [file ppat.1008822.s009.tif]

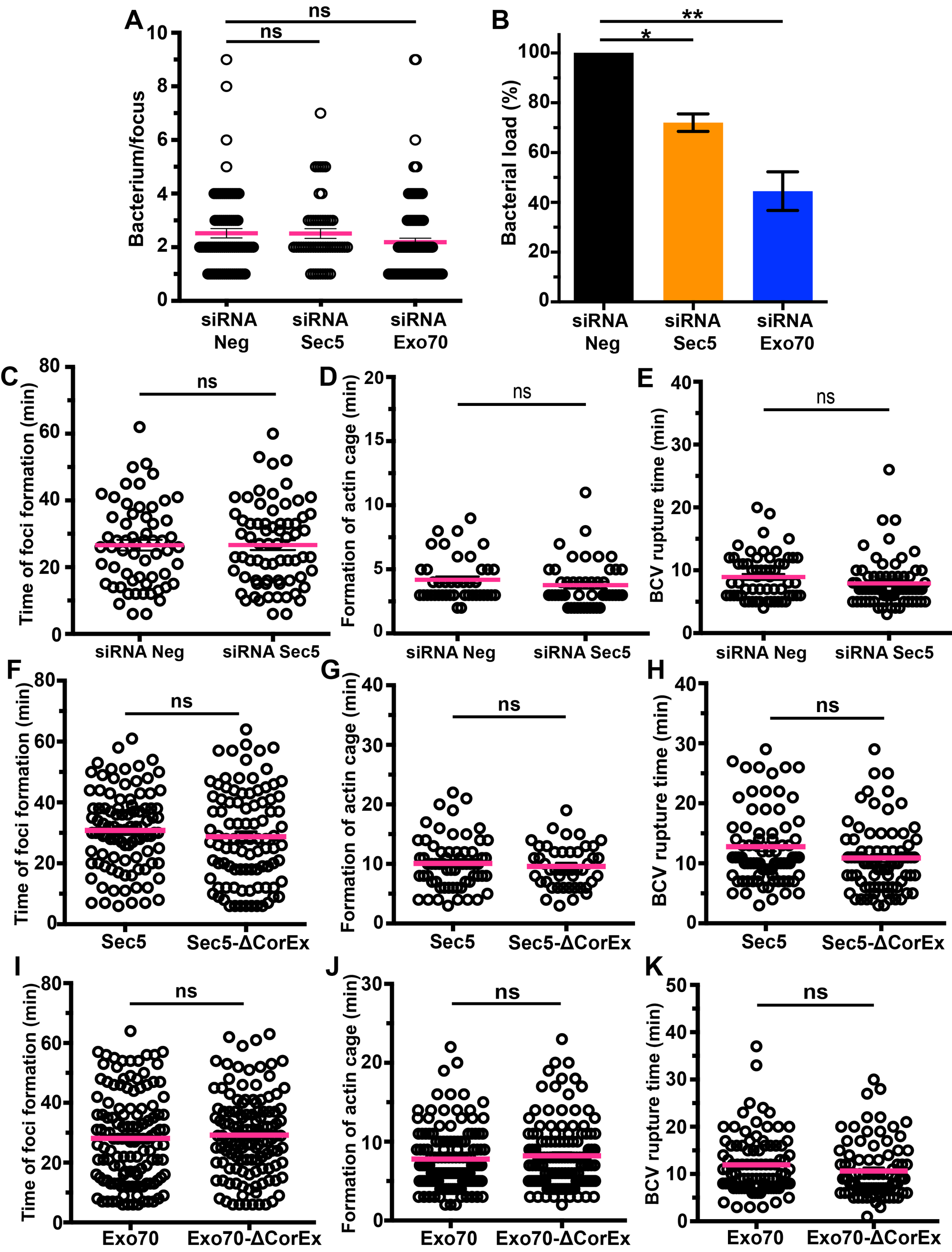

Supplement: S5 Fig — (A) Analysis of the number of bacterium per infection focus after challenging HeLa cells with S. flexneri for 30 min in Sec5- or Exo70-depleted cells or in control HeLa cells (n > 50). Data were shown as mean ± SEM (ns: non-significant). (B) Gentamicin protection assay of S. flexneri infection of Sec5- or Exo70-depleted and control HeLa cells was performed to study the effect of depleting Exo70 on the later stage of S. flexneri invasion (2hr-post infection). CFU in Exo70-depleted condition was normalized against that in HeLa control. Data were shown as mean ± SEM (n = 4) (**p<0.01; *p<0.05). Time-lapse microscopy was employed to examine the time of actin foci formation, time of actin cage formation and time of BCV rupture in RNA interference of the non-targeting control (siRNA Neg) versus Sec5-depletion (C-E), cells expressing Sec5 or Sec5 lacking the core exocyst assembly motif (Sec5-ΔCorEx) (F-H) and in cells expressing Exo70 or Exo70 lacking the core exocyst assembly motif (Exo70-ΔCorEx) (I-K). Infection foci (n > 95), actin cage (n > 45) and individual BCVs (n > 65) were analyzed in triplicates for each condition. The bars (magenta) represent the mean and unpaired t-tests were carried out (ns: non-significant). (TIF) [file ppat.1008822.s010.tif]

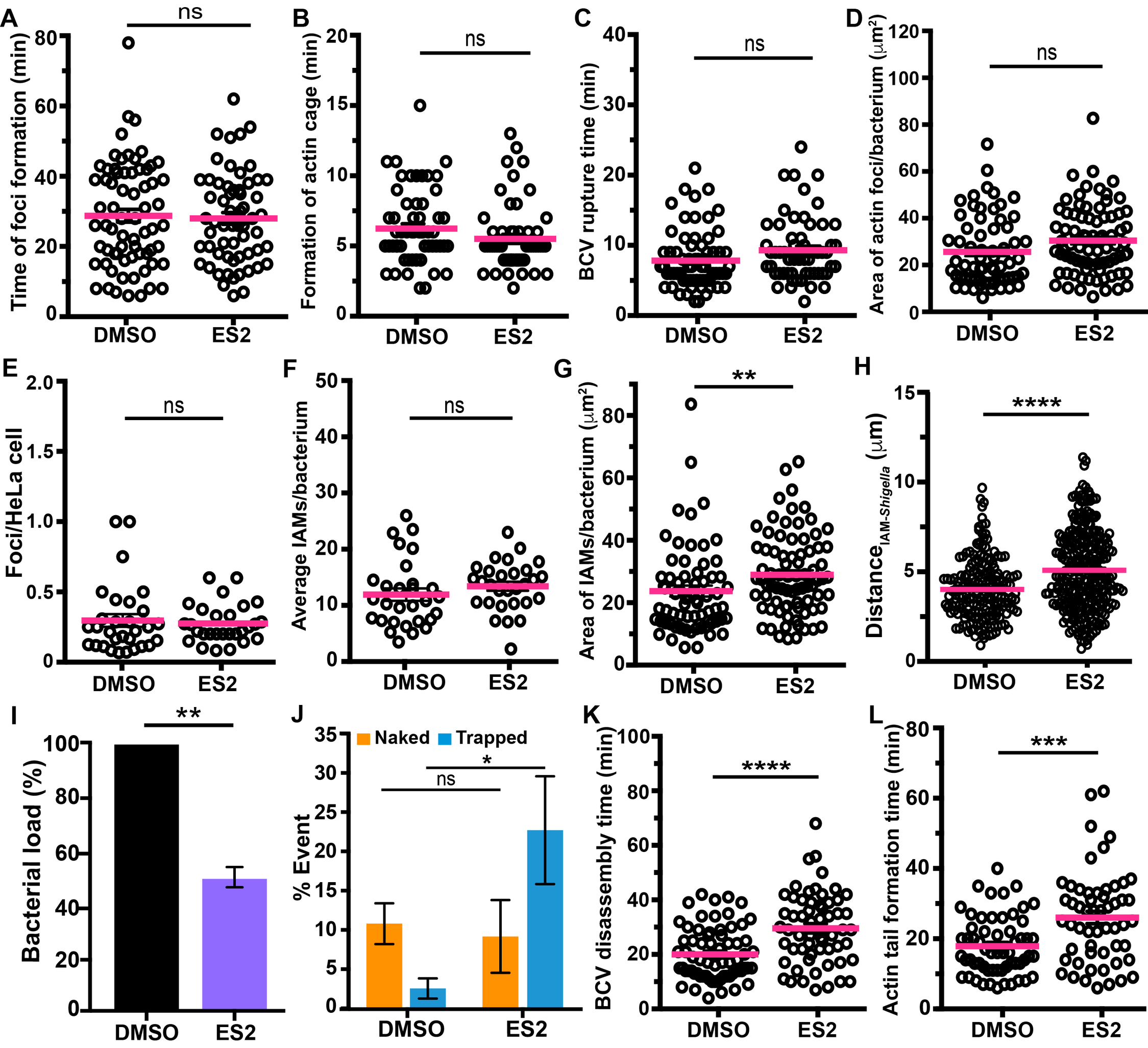

Supplement: S6 Fig — Time-lapse microscopy was employed to examine (A) the time of actin foci formation, (B) time of actin cage formation and (C) time of BCV rupture in ES2-treated and DMSO control cells. S. flexneri infection of ES2-treated and DMSO control cells in the presence of the fluorescent dextran was fixed at 30min-post infection and (D) the area of the actin foci, (E) number of infection foci per HeLa cell, (F) the number of IAMs per S. flexneri, (G) the area occupied by IAMs per S. flexneri were evaluated. Infection foci (n > 55) were examined in triplicates for each analysis. The areas occupied by IAMs were estimated by software ImageJ as illustrated in S4M Fig while (H) the distance between individual IAM and S. flexneri was estimated by software Icy in 3D as illustrated in S4N Fig. (I) Gentamicin protection assay of S. flexneri infection of ES2-treated and control HeLa cells was performed (2 hr-post infection). CFU in ES2-treated condition was normalized against that in HeLa control. Data were shown as mean ± SEM (n = 3) (**p<0.01). (J) Analysis of the fates of individual S. flexneri and their BCVs with reference to the observations in Fig 4A in ES2-treated and control cells. The data are shown in mean ± SEM (n = 3) (ns: non-significant; *p<0.05). The bars (magenta) represent the mean and unpaired t-tests were carried out (****p<0.0001). Time-lapse microscopic examination of (K) the time of BCV fragmentation and (L) formation of the actin tail in ES2-treated and DMSO control. The bars (magenta) represent the mean and unpaired t-tests were carried out (ns: non-significant; ***p<0.001; ****p<0.0001). (TIF) [file ppat.1008822.s011.tif]

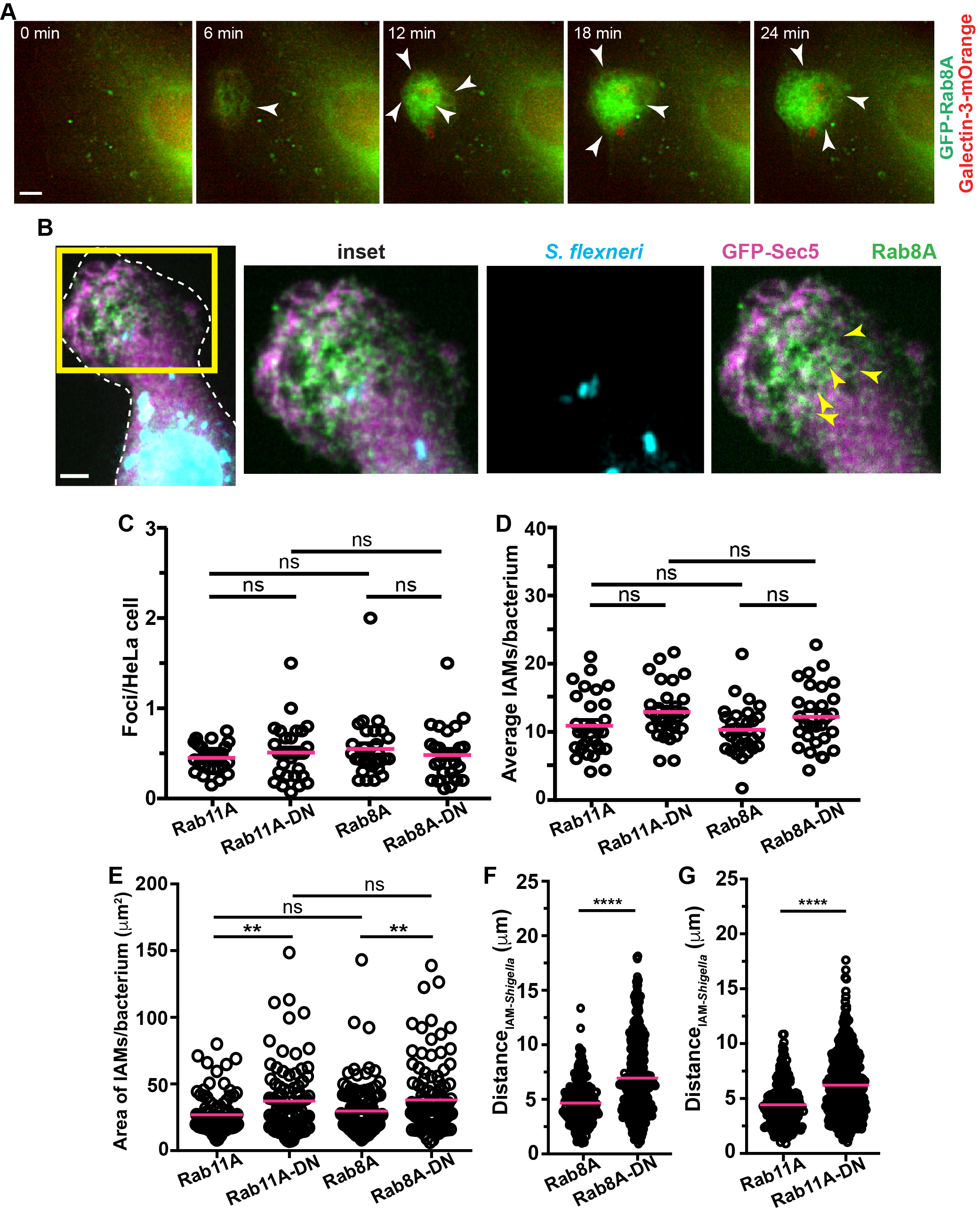

Supplement: S7 Fig — (A) Time-lapse microscopic images of the recruitment of Rab8A (green) with reference to the BCV rupture marked by Galectin-3 (red). Images were captured every minute and the z-projections of a representative infection focus were shown. The white arrowheads indicate some of the Rab8A-positive IAMs. Scale bar is 5 μm. (B) Confocal image of z-projection of a representative infection focus of S. flexneri-infected HeLa. Inset of the infection focus was highlighted in yellow and was shown, where nuclei and S. flexneri were stained by DAPI (blue). Immunofluorescence staining of endogenous Rab8A showed that GFP-tagged Sec5 colocalized with Rab8A on some IAMs (marked by the yellow arrowheads). Scale bar is 5 μm (C) Number of infection foci per HeLa cells, (D) number of IAMs per S. flexneri and (E) area occupied by IAMs per S. flexneri at individual infection foci in Rab11A, dominant negative mutant of Rab11A (Rab11A-DN), Rab8A and dominant negative mutant of Rab8A (Rab8A-DN). 29 images consisting of at least 90 infection foci (n > 90) were analyzed in triplicate experiments of each condition. The bars (magenta) represent the mean and the unpaired t-tests were carried out (ns: non-significant; **p<0.01; ****p<0.0001). Distance between individual IAM and S. flexneri was estimated in 3D by software Icy as illustrated in S4N Fig and compared in (F) cells expressing Rab8A and Rab8A-DN and (G) cells expressing Rab11A and Rab11A-DN. The bars (magenta) represent the mean and unpaired t-tests were carried out (****p<0.0001). (TIF) [file ppat.1008822.s012.tif]
