# Supplementary material for: Shigella hijacks the exocyst to cluster macropinosomes for efficient vacuolar escape
Source: PLoS Pathog. 2020 Aug 31;16(8):e1008822. doi: 10.1371/journal.ppat.1008822 (PMC7485983; doi:10.1371/journal.ppat.1008822)
Supplement: Attachment — Submitted filename: ChangEtAl_PPATHOGENS-D-20-00683_Response_to_reviewers.docx [file ppat.1008822.s021.docx]

Responses to Reviewers’ Questions

We would like to express our sincere gratitude to the Reviewers for their time to provide feedback helping us to improve the manuscript. We have taken into consideration all the suggestions and the feedback from the reviewers. Our answers to the individual comments are shown in blue in this letter.

Reviewer #1: Entry into human epithelial cells is an essential first step in shigellosis, the diseases caused by bacteria of the genus Shigella. This intracellular pathogen induces uptake of the bacterium using a Type 3 Secretion System. Soon after entry Shigella escape from the bacteria containing vacuole (BCV) and begin actin-based motility to propel themselves through the cytosol and into neighboring cells. Recent advances in imaging methods have allowed for a more detailed understanding of this process and it is now known that Shigella entry is accompanied by newly formed vesicles (macropinosomes) that come in close proximity to the BCV. Some of the molecular players involved have been identified, largely by using a “candidate gene” approach. Biochemical experiments that could identify the identity of molecules involved in an unbiased manner have been lacking.

In this fine manuscript, Chang and co-workers use a clever approach involving magnetic beads to purify newly formed vesicles (infection associated macropinosomes-IAMs) that accompany the early steps of Shigella infection. The study begins with proteomic analysis of the purified IAMs, the authors use uninfected controls and separate their membrane fractions into magnetic and non-magnetic ones. They identify a relatively tight data set of 83 proteins. These proteins are enriched for microtubule proteins and also those involved in vesicle trafficking, especially the exocyst complex. The role of the exocyst is then explored.

Live cell imaging shows that exocyst components are recruited to IAMs around the bacteria and that Sec3 and Sec5 are present right around the BCV in the moments corresponding to vacuole rupture while the exocyst component Exo70 is restricted to IAMs. Knockdown experiments that deplete Eco70 or Sec5 show that these factors are not required for IAM formation, but they act to concentrate these vesicles around the BCV. Moreover, this clustering requires the core exocyst assembly motif that is present in 2 of the proteins of interest.

Gentamicin-protection assays performed on Sec5 and Exo70 depleted cells suggest a role of exocyst in later stages of infection and this prompts the authors to carefully examine the fate of Shigella after vacuole rupture. They describe two phenotypes. The first are “naked” bacteria that rapidly break free from the BCV, which disappears and then begin actin based motility. The second population are those that remain associated with the BCV and are delayed in getting their actin based motility going. Altogether these data make a strong case for the role of exocyst in helping free Shigella to the cytosol and precisely identify the step where this occurs. Efforts are made to determine how the exocyst is recruited to the IAMs. Rab8, Rab11, and the Shigella effector IpgD are all shown to contribute to exocyst recruitment.

This study represents an important advance in the early steps of Shigella infection. The identification of novel host factors involved in this process provides new avenues to explore.

We would like to thank Reviewer #1 for the time in evaluating our manuscript and we would like to express our gratitude for the positive feedback on the novelty of the experimental approaches and the importance of our work.

Major issues:

Reviewer #1: I require no additional experiments.

We thank Reviewer #1 for this decision- in the light of the current global pandemic situation it is not easy to organize complex experimental workflows, and in the light of this we deeply appreciate this highly helpful feedback.

Minor issues:

Reviewer #1:

The text that explains the “naked” bacteria phenotype is very difficult to read. Could the authors try to make this more plain? It may help to name the non-naked bacteria phenotype (“stuck” “trapped”?).

By time-lapse microscopy, we observed that some bacteria were confined inside damaged BCVs whereas some of them were able to move away without any host membrane remnants attached. We attempted to describe our observations and classified the phenotypes according to the size of the BCV membrane remnants attached to the motile *Shigella*. More specifically, the intracellular *Shigellae* moving without any BCV membrane were termed “naked”, whereas those that moved with pieces of membrane remnants were termed “remnant” and those that moved within a perforated vacuole were termed “vacuole”. We are sorry that the text was difficult to follow. Therefore, we renamed these phenotypes, and the non-naked bacteria phenotype is now named “trapped” in the main text and in the figures. The main text has also been revised to simplify the description. We hope these changes make the manuscript easier to be followed.

The explanation of the exocyst recruitment to IAMs is fair. It is difficult to imagine these data getting more conclusive since the phenotype appears to involve both host and bacteria factors that are all pleitropic in nature.

We insisted on the unbiased experimental approaches and fair interpretation of the results. The interplay between *Shigella* and the infected host is very dynamic. We appreciate that Reviewer #1 recognized the importance as well as the challenges of our study.

The authors may consider citing the other Shigella-GBP articles that were published in the same days around their reference (#39).

We thank Reviewer #1 for the suggestion of citing the other *Shigella*-GBP articles. In fact, during this revision, more publications have been released regarding the study on GBPs on cytosolic *Shigella*. We, therefore, included these citations (Wandel *et al.*, Nat Immunol, 2020, doi: 10.1038/s41590-020-0697-2; Santos *et al.*, Nat Commun., 2020, 11, doi: 10.1038/s41467-020-16889-z).

They may also wish to discuss the early studies describing IpgD function and its role in “tethering” Niebuhr, how does this new work advance our understanding of the role of IpgD in entry (“entry” here entry used in the broad sense).

We appreciate the suggestions from Reviewer #1 on extending the discussion on the role of IpgD for *Shigella* entry. Our findings broaden the knowledge on the diverse roles of IpgD during *Shigella* invasion in addition to the reported roles in releasing the membrane tension that favors bacterial entry via cytoskeleton rearrangement (Niebuhr *et al.*, EMBO J, 2002, 21, 5069-5078) and in promoting BCV lysis (Mellouk *et al.*, Cell Host Microbe, 2014, 16, 517-530). We thus included these discussions in the main text (Discussion Section, paragraph 4).

Reviewer #2: The manuscript of Chang et al. investigates the early phase of Shigella entry into epithelial cells, the transient formation of a bacteria-containing vacuole (BCV), and the events leading of rupture of this compartment and cytosolic presence of Shigella. The Enninga group showed in early publications that interaction of the BCV with infection-associated macropinosomes (IAM) are affecting integrity of Shigella- or Salmonella-containing compartments. The present work follows a proteomic inventory of early BCV in Shigella-infected cells. An enrichment of subunits of the exocyst complex on these compartments was observed. The study further investigates the involvement of exocyst interaction of BCV and IAM by interference with exocyst subunits and specific Rab GATPases. On the bacterial side, a role of T3SS effector protein IpgD was demonstrated.

The study put focus on a very challenging event in host pathogen interaction, i.e. the transient and highly dynamic interaction of a BCV with host cell compartments between invasion and release into the cytosol. The study addresses this short and import phase by a combination of subcellular fractionation, proteomics and quantitative image analyses of live cell imaging. In addition to the technical advances provided by the work, the results provide new insight into the Shigella virulence mechanism. A novel contribution of the exocyst complex in biogenesis and rupture of BCV is provided.

The study is of high quality and provides important new results. A number of experimental issues needs further attention. The presentation of the data can also be improved.

We would like to thank Reviewer #2 for the time in carefully evaluating our manuscript. We also appreciate a lot the helpful comments on our experimental approaches and the presentation of the work.

Major issues:

Reviewer #2: A key message of the manuscript is the formation of two distinct compartments during Shigella invasion, i.e. the BCV containing Shigella, and the IAM formed as distinct entities. The infection protocol applied is critical, since magnetic beads for labeling of IAM and Shigella for invasion and formation of BCV were applied at the same time. Given the massive membrane remodelling during Shigella-mediated trigger invasion, it is rather likely that bacteria and magnetic beads are co-internalized in the same compartment. Please provide controls that distinct IAM and PCV exist prior to interaction of these entities and the rupture of the BCV. If IAM are only formed during infection, designing a clean control will be a tough cookie. As temporal dissection will not be possible, spatial separation by microscopy remains as option.

We understand the concern from Reviewer #2 and we have received similar comments previously. We have been carefully examining the *Shigella*-containing vacuole (BCV) and the infection-associated macropinosomes (IAMs) by microscopic approaches in this work and our previous work (Weiner *et al.*, PLoS Pathog, 2016, 12(5): e1005602). In our previous published work, we found by ultrastructural analysis using correlative light electron microscope (CLEM) with fluorescent dextran as the fluid-phase marker that *Shigella* never localizes within any dextran-positive vesicles, implicating that the BCV excludes most extracellular fluid during formation. Besides, we have never observed fusion between the BCV and the surrounding IAMs prior to BCV lysis during our investigations. Furthermore, we also confirmed using super-resolution structural illumination microscopy that the magnetic beads are exclusively present within the IAMs but never inside a BCV. These findings provide strong evidence that the BCV and IAMs are two distinct compartments and justify our experimental design herein in applying the magnetic beads to selectively isolate IAMs without any contamination of the *Shigella* BCV.

Along these lines, it should be considered if HeLa cells with magnetic bead only is the most suitable control. Without any stimulus for macropinocytosis, this control will be ‘super-negative’, since no macropinosomes are formed, and enrichment of INF-M over Ctrl-M has to be immense. Consider to apply an invasion-independent stimulus for macropinocytosis, followed by isolation of the resulting compartments as control for proteomics.

We thank Reviewer #2 in bringing up this concern. During our investigation in this work and our other published work (Stévenin *et al.*, Cell Rep, 2019, 29, 3958-3973), we found that the molecular composition of the IAMs are very unique to pathogen invasions. Therefore, we found it very difficult to take into consideration the stimulus-induced macropinocytosis as the experimental control because the compartments are biologically distinct from the pathogen-induced macropinosomes. Therefore, our current experimental control condition was thus chosen to be the most relevant in our model.

Along these lines, we realized that comparing the *Shigella*-induced IAMs with other pathogen-induced macropinosomes infers greater value to our proteomic analysis, which expand our knowledge on how different pathogens subvert IAMs for their intracellular niche formation. Therefore, we have initiated a comparative analysis of the proteomes from *Shigella*- and *Salmonella*-induced IAMs in our team. In response to the comments from Reviewer #2, we have included a brief discussion about the control and the above-mentioned comparative analysis in this work (Discussion section, paragraph 2).

Quantitative data (x-fold values) should be provided for the enrichment shown in Fig. 1D. The analysis was defined as quantitative proteomics, and the enrichment factors should be given for the hits that were followed up.

We appreciate the added value to include the quantitative data for the studied proteins. We have included these data as well as the results of our statistical analysis of the proteomic data as supplementary information and have deposited the raw files of the mass spectrometry experiments on the open-access PRIDE Archive (PXD020161, https://www.ebi.ac.uk/pride/archive/). In response to Reviewer #2’s comment, we have added two tables summarizing the results of our statistical analysis for the comparisons of the INF-M *vs* INF-NM and INF-M *vs* Ctrl-M (S4-S5 Tables, respectively, added to the manuscript during revision). More specifically, the enrichment (log_2_(fold-change), adjusted p-values) and label-free quantification (LFQ) intensities of the studied proteins in this work are listed. We defined that proteins are significantly enriched if they are exclusively present in the INF-M fractions in the comparative analyses or if they are found significantly enriched using a moderated t-test (FDR<1%) and with a log_2_ fold-change >1. It is thus shown in the tables that the exocyst subunits (Sec3, Sec5, Sec8, Sec15) and the regulatory Rabs (Rab8A and RalA) are significantly enriched at the IAMs in our proteomic analysis.

The quantitative image analysis workflow described in Fig. S4M is critical for the understanding of the results. However, the representation of the workflow is not fully intuitive and should be explained in more detail.

We have revised the caption of Fig. S4M and the description under the subsection “Quantitative image analysis on the distribution of the IAMs by fluorescent dextran” in the Materials and Methods section regarding the quantitative image analysis workflow. The analysis was done using the software ImageJ. In brief, we first segmented the channel of the IAMs (marked by the fluorescent dextran) from the confocal images. Next, we made a binary image of the segmented image, after which we connected the adjacent IAMs to form patches that consisted of the clustered IAMs. Last, we defined the area occupied by the IAMs at an individual infection focus as the estimated area that bordered these patches at the given focus by software Image J. The exact built-in functions in the software ImageJ for the respective steps in this protocol have already been described as well. We hope this revised description makes the workflow easier to be followed.

Minor issues:

Reviewer #2: Fig. S2AB, Fig. 2: what defines the entities highlighted by arrows as IMP? The authors used fluorescent dextran as label for live cell imaging, but a signal indicative for IMP is not registered or displayed.

In this work, we have only used the fluorescent dextran in our fixed microscopic analysis but not in live cell imaging. Hence, this is a very good remark from Reviewer #2. We have thus revised supplementary figure 2, where we had registered the IAMs by Rab11A (one of the known *Shigella* IAMs marker). The white arrows in S2A-C Fig (revised or added to the manuscript during revision) are thus marked with reference to the colocalization of the exocyst subunits on Rab11A-positive IAMs. Since we found that the IAMs are only formed upon *Shigella* infection and based on our results of the immunofluorescence staining in the fixed cell analysis in this study (S2A-C Fig, revised or added to the manuscript during revision), we can thus annotate by arrows the IAMs formed at *Shigella* infection foci in the live cell imaging.

The siRNA knock-down experiments shown in Fig. 3 and elsewhere would benefit from controls that report the overall effect on the compartment size of BCV and IAM.

We found in this work and our previous work (Weiner *et al.*, PLoS Pathog, 2016, 12(5): e1005602) that the *Shigella* BCVs do not fuse with the surrounding IAMs. Therefore, there is no change in the size of the BCV until BCV lysis, which occurs minutes after bacterial internalization. Considering the *Shigella* BCV is a very tight membrane-bound compartment enclosing the invading bacteria, the siRNA knock-down experiments thus have no effect on the size of the BCV.

In response to the comments from Reviewer #2, we analyzed the IAMs at 30-min post-infection by fluorescent dextran using software Cell Profiler (according to the protocol described in the Materials and Methods section). We found a similar trend of the sizes of the IAMs formed in the knock-down experiments compared with the control condition, confirming that knock-down of Sec5 (siRNA Sec5) or Exo70 (siRNA Exo70) exhibited no effect on the formation of the IAMs. We have included this graph showing the distribution of the size of the IAMs population in siRNA Sec5, siRNA Exo70 and in the control condition (S4P Fig, added to the manuscript during revision).

If bacterial mutant strains are used (e.g. delta ipgD), please provide evidence that the mutation can be complemented, at least by citing prior work with such strain (basic microbiology). Is the delta ipgD strain also expression afaI?

We appreciate the added value of the complementation experiment. We complemented the delta-*ipgD* mutant with a plasmid encoding the *ipgD* gene (delta-*ipgD+ipgD*) (Mellouk *et al.*, Cell Host Microbe, 2014, 16, 517-530) and performed the recruitment analysis of Exo70 and Rab11A during infection. We found that the delta-*ipgD+ipgD* strain completely rescued the recruitment of Exo70 and the colocalization of Exo70 on Rab11A-positive IAMs. We have thus revised Fig 6G to include this result in the manuscript.

The *ipgD* mutant expresses adhesin afaI as well. Based on this comment, we have revised the text in the Materials and Methods section (under subsection “bacterial strains and infection”) to specify this detail.

Page 20: adhesin afaI, not adhesion afaI

The text in the Materials and Methods section has been revised.

Page 9 and elsewhere: Western blot, not western blot

The mistakes have been corrected throughout the manuscript.

Please provide information with isoforms of Rab GTPase was analysed. In some instances, Rab8A or Rab11A are specified, but mostly only Rab5, Rab8, or Rab11 is stated.

We thank Reviewer #2 for mentioning this. The Rab GTPases studied in this work are Rab5A, Rab8A and Rab11A. We have clarified the isoforms of the Rab GTPases throughout the manuscript.

Fig. 5A is not intuitive. The orange box incorporates events that are much prior to actin tail formation.

The orange box in Fig 5A is a schematic illustration on how we define the actin tail formation time, which is the time interval between the formation of the actin cage around the BCV prior to BCV lysis and the appearance of the actin tail after vacuolar lysis. Indeed, it is our intention to depict the successive steps that constitute the vacuolar escape of *Shigella*, which is also the emphasis of this work to untangle the important steps involved in such a process for infection progression. We hope that Reviewer #2 would agree with us on keeping this figure as it is presented so as to be recapitulated in Fig 7 in order to highlight the precise discovery of this work.

In the discussion, the interactions of various intracellular pathogens with the exocytic pathway are described. The authors may wish to extend to initial and recent reports on these interactions, for example for Salmonella enterica.

In the discussion, we mentioned the diverse reported roles of the exocyst during bacterial invasion: enhancing *Salmonella* and *Staphylococcus* entry into host cells, remodeling the replicative vacuoles in *Salmonella* and *Legionella* infection and promoting *Shigella* vacuole disintegration identified in this work. We intended to discuss the subversion of the exocyst, but not the exocytic pathway, by various bacterial pathogens for their intracellular niche establishment. We appreciate the suggestion from Reviewer #2, so we have added in the discussion regarding the reprogramming of the host exocyst pathways by bacterial pathogens to remodel their replicative vacuoles (Discussion section, paragraph 3), where several studies have actually implicated the role of the exocyst in this process.

Reviewer #3: In the manuscript entitled `Shigella hijacks the exocyst to cluster macropinosomes for efficient lysosomal escape´ by Chang et al. the authors analysed the role of the cellular exocyst for Shigella infections. The authors used a proteomic approach to characterize magnetically isolated infection-associated macropinosomes (IAMs) from Shigella-infected and uninfected cells. In IAMs fraction of infected HeLa cells, proteins of the cellular exocyst including exo1, 2, 4 and 6 were significantly enriched. Next, the author applied time-lapse microscopy to analyse the localization of ectopically expressed GFP-tagged Sec3, Sec5 and Exo70 during early infection. Then the authors applied three different approaches to address the role of Sec5 and Exo70 during infections. Firstly, they used RNA interference to silence expression of these proteins. Secondly, they ectopically expressed mutated versions of Sec5 and Exo70 that fail to assemble the exocyst and thirdly, the added an inhibitor for Exo70. By these different approaches, the authors suggest novel functions of Sec5 and Exo70 for clustering of IAMs close to the bacterial containing vacuole (BCV) and for BCV disassembly that seem to help the bacterium to escape into the cytosol, a step that is essential for bacterial propagation and spread.

The manuscript is well written and presents a new approach to study IAMs in Shigella infections and suggest a novel function of the exosome for Shigella infections.
The main problem I had with the manuscript is that ectopically over-expressed tagged exocyst subunits have been shown to mislocalize in eukaryotic cells (Matern et al., 2001; Rivers-Molina et al., 2013; Ahmed et al., 2018). Thus, conclusions based on this method (e.g. Figure 1 and Figure 6) could be misleading.

In response to the general comments from Reviewer #3, we appreciate a lot Reviewer #3 for spending time in giving us very constructive and critical feedback. We also thank Reviewer #3 in recognizing the novelty of our findings and the careful evaluation of the methodologies employed in this work. We took into consideration the worries and suggestions, and as a result we have performed additional experiments. We hope that our additional work convinces Reviewer #3 that our conclusions are drawn based on solid evidence and circumspection.

Major issues:

Reviewer #3: (1) Ectopic expression: Exocyst subunits were systematically tagged with GFP in MDCK cells a decade ago, but most GFP fusions produced diffuse cytosolic labeling, incongruent with localization to vesicles (Matern et al., 2001; Rivers-Molina et al., 2013; Ahmed et al., 2018). The author must overcome this obstacle. One possibility to avoid this problem was published by Rivers-Molina et al., 2013. In this paper, Sec8 subunit was silenced by RNA interference and replaced with a tagged Sec8 version. The authors must perform new experiments as outlined above to analyse the localization of the exocyst subunits and the colocalization of exocyst subunits with Rab GTPases during infection.

We thank Reviewer #3 in raising this concern. We had anticipated such reservation, so we have performed staining of the endogenous proteins showing the enrichment of the exocyst subunits at the IAMs (original S2A-B Figs). Besides, the GFP-tagged plasmids used in this work have also been extensively used and published by other researchers (Zhao *et al.*, Dev Cell, 2013, 26(3), 266-278; Zhang *et al.*, PNAS, 2016, 113(1), E41-50). Moreover, Ahmed *et al.* validated that the GFP-tagged exocyst subunits display “substantial diffuse cytoplasmic localization” even at an expression close to the physiological level. Therefore, our work is in line with the previous published literature. Despite this, we decided to follow up more on these comments from Reviewer #3 and we tried the experimental approach described by Rivera-Molina and Toomre, J Cell Biol, 2013, 201(5), 673-680. We have carefully performed the analysis of Exo70, the most discussed exocyst subunit in this work, based on the protocol from Rivera-Molina *et al.* as a “proof-of-concept” approach to validate its localization and recruitment during *Shigella* infection with reference to the Rab11A and Rab8A.

In brief, Exo70 was knocked-down by siRNA in HeLa cells and different concentrations (0, 0.1, 0.2 and 1 μg) of the siRNA-resistant GFP-tagged Exo70 plasmid were subsequently transfected to the knock-down cells. We first confirmed the efficiency of the knock-down and checked the expression profile of Exo70 under these conditions by Western blot (Panel A of the figure below). We found that the level of GFP-Exo70 expression was comparable to the control cells at a complementation of 0.2 μg of GFP-Exo70 plasmid (Panel A of the figure below). We thus complemented 0.2 μg of plasmid encoding GFP-Exo70 to the siRNA Exo70-treated cells and performed time-lapse imaging according to the same experimental procedures as in Fig 2C. We examined the recruitment of GFP-Exo70 during *Shigella* infection with reference to the Gal3-mOrange, the BCV rupture marker used in this study. We also examined the colocalization of GFP-Exo70 with Rab8A and Rab11A during *Shigella* infection under the same experimental procedures as in Fig 6A-B.

We summarized the results in the figure below. Panel A showed the Western blot analysis of the knock-down efficiency and the expression level of GFP-tagged Exo70 at different concentrations of the complementation plasmid. Panel B-D are time-lapse microscopic images showing the recruitment of Exo70 (green) in the presence of (B) BCV rupture marker Galectin-3 (red), (C) Rab8A (red) and (D) Rab11A (red). The scale bars are 5 μm. The white arrows in panel B-D indicate the localization of Exo70 on IAMs while the magenta arrowheads mark the intracellular *Shigella*. We found that Exo70 was recruited to *Shigella* IAMs prior to BCV lysis (marked by the appearance of the Galectin-3-mOrange signal) (Panel B of the figure below), similar to result in Fig 2C. We also confirmed the colocalization of GFP-Exo70 on Rab8A- and Rab11A-positive IAMs during *Shigella* infection, recapitulating our results in Fig 6A-B ((Panel C-D of the figure below).

Apart from that, we appreciate the added value of investigating the localization of the endogenous proteins. We thus performed immunostaining of the endogenous Sec3, Sec5, Exo70 at 30 min-post infection and investigated their localizations with reference to the known *Shigella* IAM marker, Rab11A. We found that Sec3, Sec5 and Exo70 were enriched at the *Shigella* infection foci and colocalized with some Rab11A-positive IAMs (indicated by the white arrows). We have thus revised the supplementary figure and included these results in S2A-C Figs.

Collectively, these investigations substantiated our findings of the localization of the exocyst subunits to *Shigella* IAMs. Our analyses on the endogenous and the GFP-tagged proteins clearly validated that the exocyst subunits are recruited to *Shigella* IAMs, which was mediated by Rab11A, during *Shigella* infection. We have carefully thought of how to add the described additional data (that reiterate our previous data and interpretations) in our manuscript, but we could not find a good spot without interrupting the flow of the manuscript. Therefore, we hope that the Reviewer is fine having this data only added as information to the Reviewers and editor.

(2) The authors identified several proteins enriched in the IAMs fraction from infected cells. The author must validate these enriched proteins using a different method e.g staining of isolated IAMs with antibodies specific for EXOC1, EXOC2, EXOC 4, EXOC6, RAB8A. IAMs isolated from mock-infected cells should be used as controls.

We understand the concern of Reviewer #3. We thus addressed to the comments from Reviewer #3 by immunostaining the endogenous proteins. We confirmed that EXOC1/Sec3 (S2A Fig, added to the manuscript during revision), EXOC2/Sec5 (S2B Fig, added to the manuscript during revision) and Rab8A (S7B Fig) were recruited at *Shigella* IAMs, validating the proteomic results on the enrichment of these proteins on the isolated vesicles.

On the other hand, our team is performing a pairwise comparative analysis of the IAMs isolated from *Shigella* (this work) and *Salmonella* infection (Stévenin *et al.*, Cell Rep, 2019, 29, 3958-3973). We discovered that EXOC1, EXOC2, EXOC4, EXOC6 and Rab8A were only significantly enriched in *Shigella* IAMs proteome, implicating the specificity of the enrichment of these proteins on the IAMs for an important functional role in *Shigella* infection. We have integrated a discussion of this finding in this current work (Discussion Section; paragraph 2). We hope we could convince Reviewer #3 on the validity and importance of our *Shigella* IAMs proteome.

(3) Sec5 and Exo70 localize to different structure during infection. Do the authors have evidence that the exocyst is formed during infection? Could both subunits act independently and control different processes? Some more data on this is needed to better understand the role of the exocyst during Shigella infection.

The assembly of the fully functional exocyst complex has been controversial. A recent study using high-speed TIRF microscopy proposes a model for the dynamic assembly and disassembly of the exocyst complex (Ahmed *et al.*, Nat Commun, 2018, 9, 5140). Indeed, Ahmed *et al.* and other researchers (Rivera-Molina and Toomre, J Cell Biol, 2013, 201(5), 673-680) reveal that the assembly and disassembly of the exocyst complex occurs in seconds. This extremely rapid event implies a critical requirement of dedicated effort to address this precise biological event, which is beyond the biological relevance of our current work to study the vacuolar escape of *Shigella* and bacterial invasion. Therefore, we did not pursue the study on the formation of the exocyst complex in this current work.

By time-lapse microscopic analysis (Fig 2D), we found that Sec5 (one component of the exocyst subcomplex I) was strongly recruited to *Shigella* IAMs and in close proximity of the BCV (Fig 2B), while Exo70 (one component of the exocyst subcomplex II) is only present strongly at the IAMs (Fig 2C). This observation thus encouraged us to construct the mutant proteins that lack the core exocyst assembly motif (Sec5-ΔCorEx and Exo70-ΔCorEx) to study the effect of interrupting the assembly of the exocyst complex. Our findings indicate that the expression of Sec5-ΔCorEx or Exo70-ΔCorEx resulted in a more dispersed spatial distribution of the IAMs (Figs 3C-D and Figs 3G-H, respectively), which is comparable to the effect of knock-down of Sec5/Exo70. We thus concluded that the exocyst complex and potentially the assembly of the fully functional complex are involved in clustering IAMs in the close proximity to intracellular *Shigella*. Further studies are warranted to investigate if the exocyst subunits are recruited to the IAMs to act on many different processes besides IAM-clustering revealed by us. Nevertheless, our work signifies the first report to elucidate the molecular mechanism of the subversion of the host trafficking pathway (involving the exocyst complex and its regulatory Rab GTPases) in facilitating the escape of an invasive pathogen from its vacuole.

Minor issues:

Reviewer #3: (1) Abstract: the authors stated that `proteins […] were significantly enriched at the IAMs […]´. What is the evidence for this? It appears that these proteins are enriched in the fraction of IAMs isolated from infected cells. Please clarify.

We have included the quantitative data of our proteomic analysis in the manuscript. For this, we have added two tables summarizing the proteins found significantly regulated from the statistical analyses INF-M *vs* INF-NM and INF-M *vs* Ctrl-M (S4-S5 Tables, respectively, added to the manuscript during revision) for the studied proteins. We stated the enrichment (log_2_(fold-change), adjusted p-values) and label-free quantification (LFQ) intensities of the studied proteins in the tables for the triplicate experiments. In our work, we defined the proteins as “significantly enriched” if they are exclusively quantified in the INF-M fractions in the comparative analyses, or if the proteins are found significantly enriched using a moderated t-test (FDR<1%) and have a log_2_(fold-change)>1. It is shown that the proteins are enriched in the fraction containing the isolated IAMs from the infected cells (INF-M fraction). We thus concluded that the exocyst subunits (Sec3, Sec5, Sec8, Sec15) and the regulatory Rabs (Rab8A and RalA) are significantly enriched at the IAMs in our proteomic analysis.

(2) Are magnetic beats in uninfected cells as efficiently taken up as in infected cells?

Taking the advantages of the fluorescent dye coated on the surface of the magnetic beads, we analyzed the different fractions after each magnetic purification using a fluorimeter. After calibration and producing a standard curve using different concentrations of the magnetic beads, we evaluated the “extraction efficiency” of each purification. We found that on an average of triplicate experiments, ~3-fold more beads were retained in the magnetic fraction of the infection samples (INF-M) than those from the uninfected cells (Ctrl-M). Therefore, the magnetic beads are not taken up efficiently by the treated cells in the absence of the bacteria, however there is some background stickiness.

(3) Why did the authors focus on Rab8A while Rab9 was significantly enriched in INF-M factions?

In our proteomic analysis, we defined a protein candidate to be significantly enriched if they are exclusively present in the INF-M fractions in the comparative analyses or if the proteins are found significantly enriched using a moderated t-test and have a log_2_(fold-change)>1. By this definition, in fact, both Rab8A and Rab9A are considered to be significantly enriched in the INF-M fractions. We decided to focus the study on Rab8A rather than Rab9 in this work because we found a significant enrichment of proteins involved in the recycling and exocytic pathway at the IAMs, particularly the different subunits of the exocyst complex and the regulatory GTPase Rab8A. This is of particular interest to us because we have demonstrated previously a functional role of Rab11 on the IAMs during *Shigella* invasion (Mellouk *et al.*, Cell Host Microbe, 2014, 16, 517-530; Weiner *et al.*, PLoS Pathog, 2016, 12(5): e1005602). We thus focused our effort on elucidating the role of the exocyst, together with the regulatory Rab GTPases Rab8A and Rab11A, on *Shigella* vacuolar escape.

(4) The author found Exo70 in IAMs by microscopy. Why has Exo70 not been identified in the proteomic fraction?

We would like to emphasize that Exo70 was identified from our mass spectrometry experiments but with only four different unique peptides among all the samples using our data analysis pipeline (MaxQuant/Andromeda search engine, see Materials and Methods section). Of note, at least one unique peptide of this protein has been quantified in all fractions (INF-M, INF-NM and Ctrl-M), showing it is present in our experiments (but with very low abundance). However, to obtain a relative abundance value for a protein so that we can confidently compare its abundance between two fractions, we applied the default parameters of the quantification algorithm provided by MaxQuant to get LFQ intensities, i.e. at least 2 quantified ‘*unique+razor’* peptides are needed to get a LFQ value for a protein in a sample. Unfortunately, as we detected too few peptides for Exo70, this condition was checked in only one replicate of the INF-NM fraction, while no LFQ values were quantified in the INF-M and Ctrl-M fractions. According to our selective criteria and parameters, there were therefore not enough LFQ values for Exo70 to allow the quantification of this protein. Indeed, to ensure a minimum of replicability, a protein has been quantified and included in our analyses if it has been quantified in at least two samples of one of the studied fractions. Therefore, we can confirm the presence of Exo70 on the IAMs and in the proteomic fraction by mass spectrometry but in too low abundance to be confidently quantified by using our pipeline and its abundance to be compared across the different fractions.

Although we do not obtain sufficiently reliable data to conclude that Exo70 is significantly enriched in the INF-M fraction from our proteomic analysis, we demonstrated by fixed and time-lapse microscopy that Exo70 was present at the IAMs at endogenous condition (S2C Fig & Fig B-D for addressing “Major concern” – Question 1 in this letter). Moreover, our analyses using different experimental approaches evidently propose a functional role of Exo70 (hence the exocyst complex) in the vacuolar escape of *Shigella*.

(5) Please confirm knockdown efficiency by western blotting.

We took into consideration of the comment from Reviewer #3 and have checked the knock-down efficiency of Exo70 and Sec5 by Western blotting. We found that both Exo70 (S3B Fig) and Sec5 (S4O Fig, added to the manuscript during revision) were efficiently knocked-down in our tested condition stated in the Materials and Methods section.

(6) How did the authors define close proximity?

In our time-lapse microscopy, we observed many instances where the GFP-tagged Sec3 and Sec5 bordered the BCV that has been marked by the rupture marker Galectin-3 (Fig 2A-B). It is indeed tempting to claim the “colocalization” of these proteins on the BCV or on BCV membrane remnants from these observations. However, taking into consideration the resolution limits of the microscopic techniques employed in this work, we carefully described these observations as localization of these proteins to the “close proximity” to the invading bacteria to avoid overstating the observations.

(7) What is the fate of entrapped bacteria in Exo70 and Sec5 silenced cells?

In this work, we found that *Shigella* has a higher probability to be entrapped in the damaged vacuoles and a reduced actin-based motility in the Sec5 or Exo70 knock-down conditions. We reason that the infection progression of these bacteria is thus significantly impaired and will be more prone to degradation by the infected host. One possible clearance mechanism is the targeting of the cytosolic *Shigella* by guanylate-binding proteins (GBPs) that initiates the capase-4 activation and the host immune defense pathways (Wandel *et al.*, Nat Immunol, 2020, doi: 10.1038/s41590-020-0697-2; Santos *et al.*, Nat Commun., 2020, 11, doi: 10.1038/s41467-020-16889-z).

(8) Does the inhibitor ES2 affect bacterial propagation?

Yes. We checked the effect of inhibitor ES2 on the bacterial propagation by Gentamicin protection assay, where we pre-treated HeLa cells with ES2 (40 μM) for 4 hr as described previously (Zhang *et al.*, PNAS, 2016, 113(1), E41-50) and examined the intracellular bacterial load 2 hr-post infection. We found that the intracellular bacterial load decreased to about 50% of the control condition after ES2 treatment, indicating that disrupting the function of Exo70 by specific inhibitor ES2 inhibited *Shigella* propagation (S6I Fig, added to the manuscript during revision). This effect is similar to the interruption of the function of Exo70 by RNA interference.

(9) There are few sentences that are difficult to understand e.g. page 9, 1st paragraph, last sentence. Please re-write.

We thank the comments from the Reviewer #3. We have revised some sentences in the manuscript. We hope Reviewer #3 would find the revised version easier to read.
